# False positive metastatic disease due to combined thoracic and subcutaneous splenosis

**DOI:** 10.1016/j.radcr.2023.11.050

**Published:** 2023-12-15

**Authors:** Jeffrey Chen, Robert Russo, Grace Yung, Clarence Yeong, Robert Mansberg

**Affiliations:** a Department of Molecular Imaging Concord Hospital, Concord, NSW, Australia; b Department of Respiratory and Sleep Medicine, Concord Hospital, Concord, NSW, Australia; c Faculty of Medicine and Health, University of Sydney, NSW, Australia

**Keywords:** False positive metastatic disease, FDG PET, Thoracic and subcutaneous splenosis, Heat damaged red blood cell scan

## Abstract

A 56-year-old man presented with dyspnea secondary to pulmonary emboli and dilated cardiomyopathy. His past medical history included a history of emergency laparotomy, splenectomy, and splenic flexure resection following a gunshot injury 30 years ago. CT and MRI imaging demonstrated multiple homogeneously enhancing lobulated lesions at the left-sided pleura and chest wall with an irregular calcified spleen. The aforementioned lesions demonstrated a similar level of tracer uptake to the splenic activity with no evidence of other FDG avid malignancy on the follow-up ^18^F-FDG PET study. All the above-mentioned pleural and chest wall lesions demonstrated intense tracer accumulation on technetium-99m labeled heat-damaged red cell scintigraphy, consistent with combined thoracic and subcutaneous splenosis.

## Introduction

Splenosis is the heterotopic transplantation of splenic tissue to ectopic sites, which was first described by Buchbinder and Lipkoff in 1939 [1]. Splenosis typically occurs following traumatic or surgical splenic rupture, with a median interval of 20 years between the splenic injury and diagnosis of splenosis [2]. Splenosis has been reported to be detected in up to 67% of patients with a history of splenic rupture [3]. Most splenosis lesions are identified intraperitoneally at the splenic bed, while thoracic splenosis is the most common extraperitoneal site following simultaneous splenic and diaphragmatic injury [4]. Subcutaneous splenosis is extremely rare, with most reported subcutaneous splenosis identified at the abdominal surgical scars [5].

Due to its low prevalence, diagnosing thoracic and subcutaneous splenosis through noninvasive methods is challenging, especially when the mass presents as a malignant disease on imaging as in this case. Hence, diagnosing thoracic and subcutaneous splenosis remains elusive and warrants further investigation, in particular through Technetium-99m labeled heat-damaged red cell scintigraphy.

## Case presentation

**Fig. 1:**
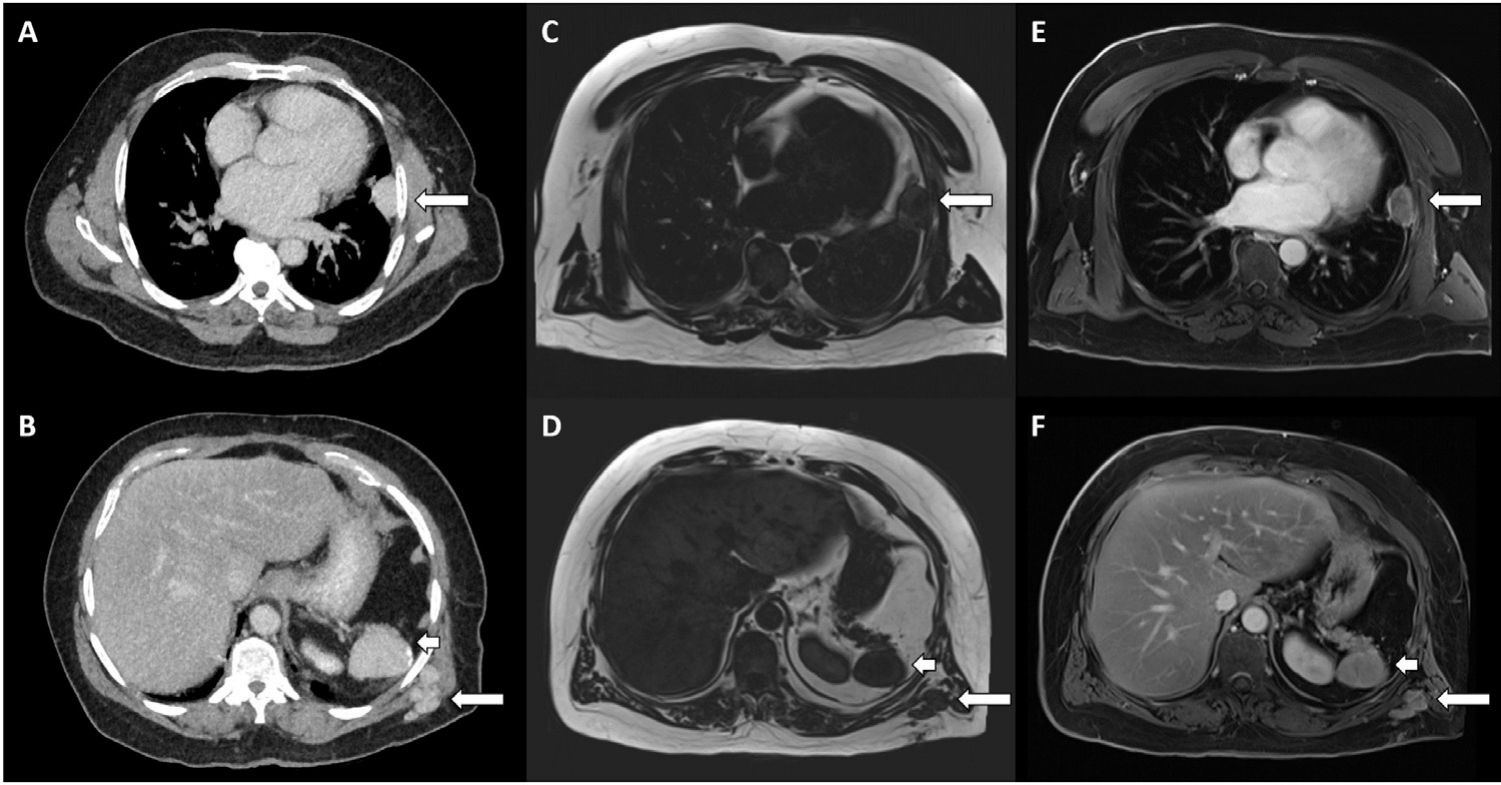
A 56-year-old man was admitted to the hospital for management of bilateral pulmonary emboli and investigation of dilated cardiomyopathy. His past medical history revealed a history of gunshot injury to the left chest requiring emergency laparotomy 30 years ago. IV-contrast enhanced CT imaging demonstrated homogeneously enhancing lobulated lesions at the left-sided pleura (A, long arrow) and left posterolateral chest wall (B, long arrow) with irregular partially-calcified spleen (B, short arrow). On Gadolinium-enhanced MRI imaging, the pleura and chest wall lesions demonstrated similar signal intensity and enhancement pattern to that of the spleen (D and F, short arrows), with central low signal intensity and hypointense rim on T1-weighted imaging (C and D, long arrows), and homogeneous enhancement on post-Gadolinium imaging (E and F, long arrows).

A 56-year-old man was admitted to the hospital for management of bilateral pulmonary emboli and investigation of dilated cardiomyopathy. His past medical history revealed a history of gunshot injury to the left chest requiring emergency laparotomy 30 years ago. IV-contrast enhanced CT imaging demonstrated homogeneously enhancing lesions at the left-sided pleura (Fig. 1A, long arrow) and left posterolateral chest wall (Fig. 1B, long arrow) with irregular partially-calcified spleen (Fig. 1B, short arrow). The pleura and chest wall lesions demonstrated similar signal intensity and enhancement pattern to that of the spleen on Gadolinium-enhanced MRI imaging (Figs. 1C–F).

**Fig. 2:**
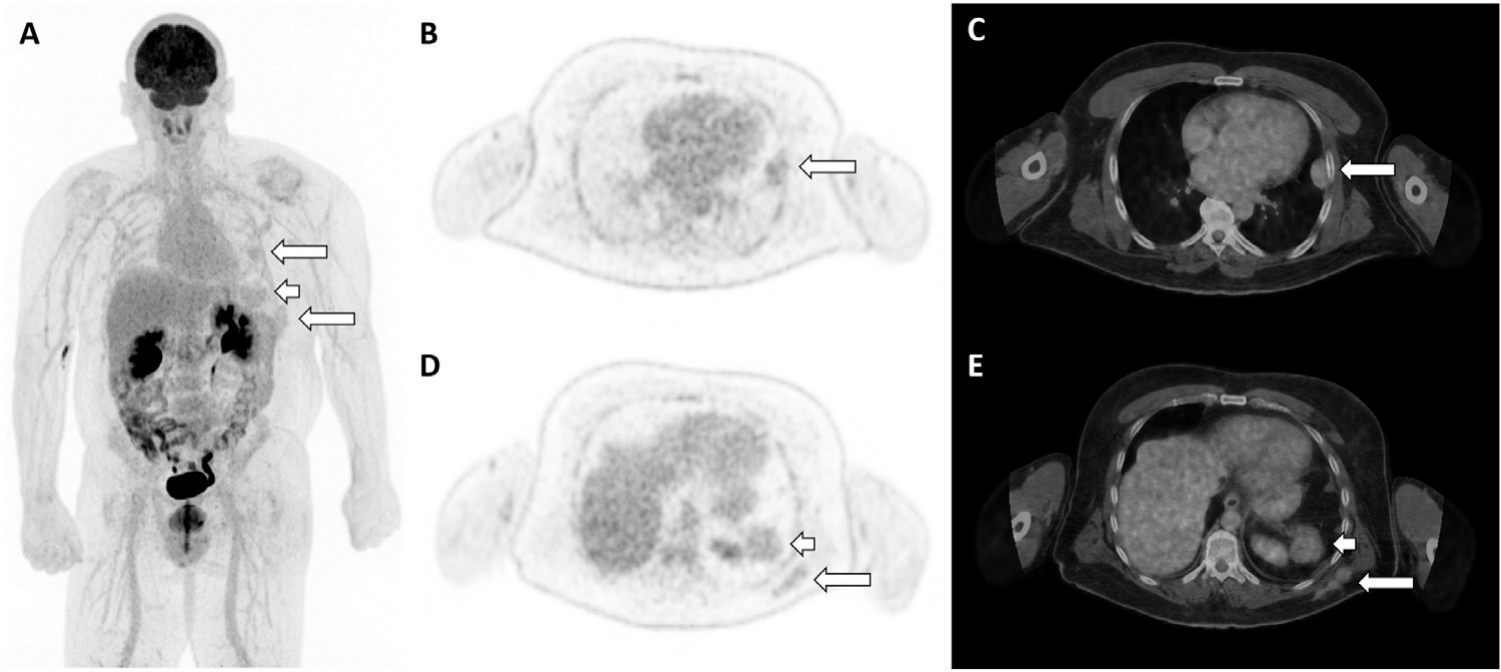
^18^F-FDG PET Maximal intensity projection (A), axial FDG PET (B and D) and axial hybrid imaging (C and E) demonstrated low-grade FDG avidity (SUVmax up to 2.6; long arrows) at the pleural and subcutaneous lesions, similar to background splenic activity (SUVmax 3.0; short arrows).

^18^F-FDG PET study was subsequently performed to investigate possible malignancy. Maximal intensity projection (Fig. 2A), axial FDG PET (Figs. 2B and D), and axial hybrid imaging (Figs. 2C and E) demonstrated low-grade FDG avidity (SUVmax up to 2.6; long arrows) at the pleural and subcutaneous lesions, similar to background splenic activity (SUVmax 3.0; short arrows).

**Fig. 3:**
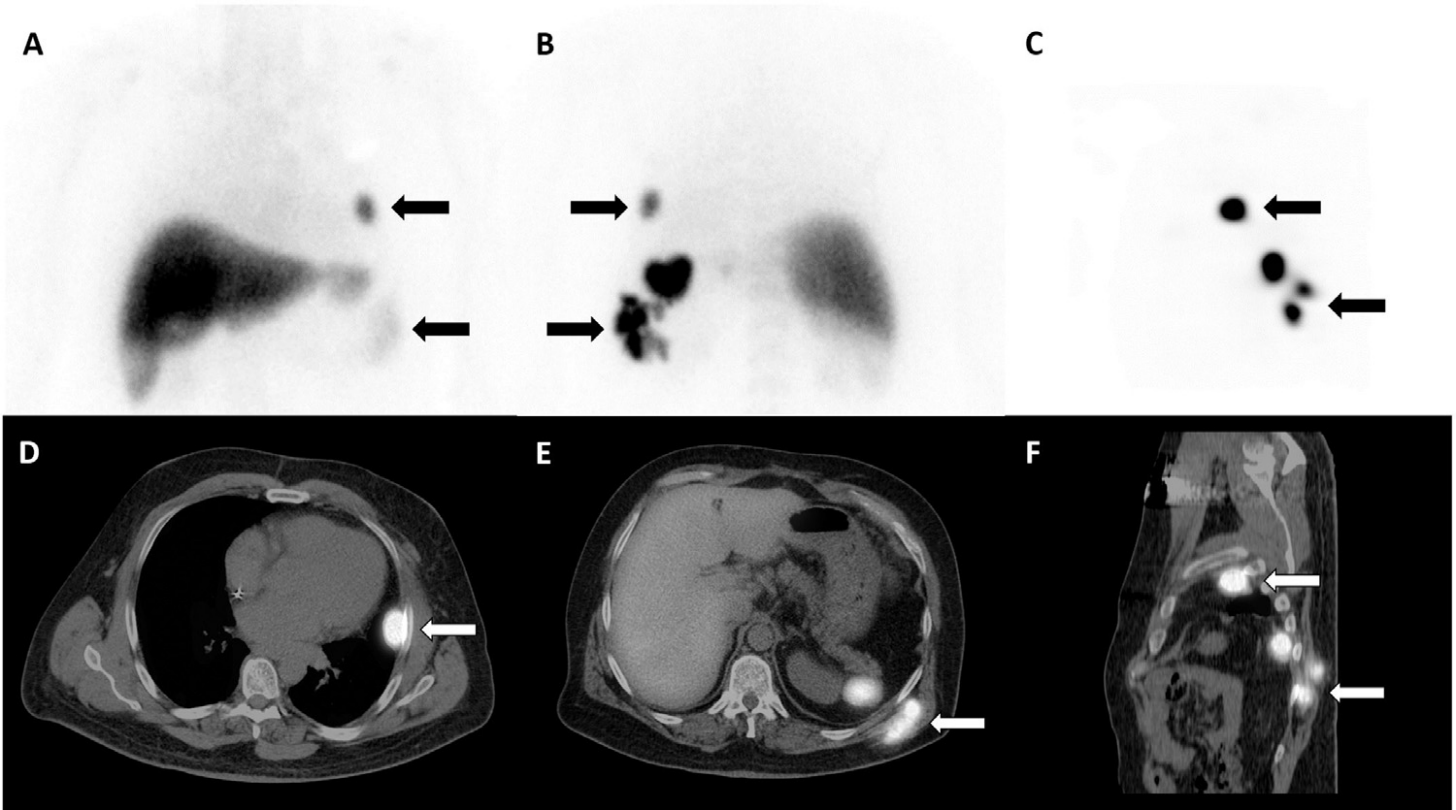
Technetium-99m labeled heat-damaged red cell scintigraphy anterior-posterior planar imaging and sagittal SPECT images demonstrated intense tracer activity in the region of the left hemithorax and left chest wall (A–C; Black arrows). Hybrid axial and sagittal SPECT/CT images confirmed extra-splenic tracer accumulation at the left pleural and left chest wall lesions, confirmed the diagnosis of combined thoracic and subcutaneous splenosis (D and E; White arrows).

Given similar imaging characteristics and FDG avidity of the pleural and subcutaneous lesions to that of the spleen, the patient was referred for Technetium-99m labeled heat-damaged red cell scintigraphy to assess for splenosis. The planar images demonstrated intense tracer activity in the region of the left hemithorax and left chest wall (Figs. 3A–C; Black arrows). Hybrid SPECT/CT images confirmed extra-splenic tracer accumulation at the left pleural and left chest wall lesions, and confirmed the diagnosis of combined thoracic and subcutaneous splenosis (Figs. 3D and E; White arrows).

## Discussion

Splenosis is the heterotopic splenic tissue typically resulting from traumatic or surgical splenic rupture. The pathogenesis of autotransplantation is postulated to be related to the seeding of splenic pulp into the surrounding tissue [5], while the second proposed mechanism is the hematogenous spread of splenic pulp as postulated in case reports of cerebral and intrahepatic splenosis [6,7]. Clinically, symptomatic thoracic splenosis cases may present with pleuritic chest pain and hemoptysis, while intra-abdominal splenosis may develop complications such as hemorrhage and local mass effect. However, splenosis is typically asymptomatic and identified as an incidental mass which may mimic malignancy as in this case.

Splenosis deposits demonstrate similar imaging characteristics to the native spleen across various imaging modalities. In CT imaging, splenosis appears as round lobulated lesions with identical enhancement characteristics to that of the normal splenic tissue. In MRI, splenosis nodules exhibit homogeneous central hypointensity with a hypointense rim on T1- and T2-weighted imaging, with enhancement patterns resembling that of the spleen [8,9]. However, in the absence of clinical history and given the rarity of the condition, splenosis is often confused with neoplastic lesions, leading to unnecessary invasive investigation and surgery.

Technetium-99m labeled heat-damaged red cell scintigraphy is currently the diagnostic modality of choice for splenosis utilizing the sequestration of damaged red blood cells in the reticuloendothelial cells within the splenosis tissue, and has proven to be highly sensitive and specific for splenosis [10,11]. Therefore, nuclear scintigraphy can noninvasively confirm the diagnosis of splenosis which can be managed conservatively and limits unnecessary surgical treatment.

## Patient consent

Written informed consent was obtained
